# Genetic mutation and tumor microbiota determine heterogenicity of tumor immune signature: Evidence from gastric and colorectal synchronous cancers

**DOI:** 10.3389/fimmu.2022.947080

**Published:** 2022-11-07

**Authors:** Weili Yang, Yaxing Zhao, Qiongxiang Ge, Xiaoli Wang, Yang Jing, Jingwen Zhao, Gang Liu, He Huang, Fei Cheng, Xiaoxi Wang, Yulin Ye, Wenjing Song, Xinjuan Liu, Juan Du, Jianpeng Sheng, Xiaocang Cao

**Affiliations:** ^1^ Department of Gastrointestinal Surgery, The First Affiliated Hospital, Zhejiang University School of Medicine, Hangzhou, China; ^2^ Department of Hepatobiliary and Pancreatic Surgery, The First Affiliated Hospital, Zhejiang University School of Medicine, Hangzhou, China; ^3^ Zhejiang Provincial Key Laboratory of Pancreatic Disease, The First Affiliated Hospital, Zhejiang University School of Medicine, Hangzhou, China; ^4^ Zhejiang University Cancer Center, Zhejiang University, Hangzhou, China; ^5^ Department of Anorectal Surgery, The First Affiliated Hospital of Zhejiang Chinese Medical University, Zhejiang Provincial Hospital of Traditional Chinese Medicine (TCM), Hangzhou, China; ^6^ Department of Hepato-Gastroenterology, Tianjin Medical University General Hospital, Tianjin Medical University, Tianjin, China; ^7^ Department of Surgery, Tianjin Medical University General Hospital, Tianjin Medical University, Tianjin, China; ^8^ Frontiers Science Center for Synthetic Biology, School of Chemical Engineering and Technology, Tianjin University, Tianjin, China; ^9^ Department of Pathology, The First Affiliated Hospital, Zhejiang University School of Medicine, Hangzhou, China; ^10^ Department of Pathology, Tianjin Medical University General Hospital, Tianjin Medical University, Tianjin, China; ^11^ Department of Gastroenterology, Beijing Chaoyang Hospital, Capital Medical University, Beijing, China; ^12^ Department of Gastroenterology, First Affiliated Hospital of Zhejiang University School of Medicine, Hangzhou, China

**Keywords:** single-cell RNA sequencing, microbiome, whole-exome sequencing, colorectal cancer, gastric cancer

## Abstract

Both colorectal and gastric cancer are lethal solid-tumor malignancies, leading to the majority of cancer-associated deaths worldwide. Although colorectal cancer (CRC) and gastric cancer (GC) share many similarities, the prognosis and drug response of CRC and GC are different. However, determinants for such differences have not been elucidated. To avoid genetic background variance, we performed multi-omics analysis, including single-cell RNA sequencing, whole-exome sequencing, and microbiome sequencing, to dissect the tumor immune signature of synchronous primary tumors of GC and CRC. We found that cellular components of juxta-tumoral sites were quite similar, while tumoral cellular components were specific to the tumoral sites. In addition, the mutational landscape and microbiome contributed to the distinct TME cellular components. Overall, we found that different prognoses and drug responses of GC and CRC were mainly due to the distinct TME determined by mutational landscape and microbiome components.

## Introduction

Both colorectal and gastric cancers are aggressive solid-tumor malignancies, leading to the second and third most common causes of cancer-associated deaths worldwide (1, 2). Colorectal cancer (CRC) and gastric cancer (GC) share many similarities, possibly due to their similar origin of intestinal epithelial cells. Both CRC and GC display comparable progression patterns, from the incidence of submucosal invasion and lymphatic infiltration to lymph node metastasis (3). CRC and GC also show a similar genetic landscape. For example, APC mutation is often identified in both CRC and GC (4). Molecular features are also identical between CRC and GC, e.g., an overactivated KRAS signaling pathway can be detected (4, 5).

Although CRC and GC share many similarities, they also display distinct prognoses, drug response to chemo, and immunotherapy. The 5-year survival rate for GC patients with localized disease is 68.6%, while the 5-year survival of GC patients with advanced disease is only 5.3% (2). Meanwhile, the 5-year survival rate for CRC is about 64% but drops to 12% for advanced CRC patients (6). Chemotherapy is the major option for CRC and GC patients nowadays. Current chemotherapy includes single-agent therapy, which is mainly fluoropyrimidine (5-FU), and multiple-agent regimens including oxaliplatin (OX), irinotecan (IRI), and capecitabine (CAP or XELODA or XEL) (6). CRC and GC responses to chemotherapy are also different. FOLFOX (5-FU+OX) chemotherapy in patients with advanced CRC has increased their overall survival (OS) time to almost 20 months (7).

In contrast, gastric cancer responded poorly to the FOLFOX regime. Regarding OS of patients with advanced gastric cancer, the FOLFOX regime only increased the patients’ OS to 11 months only (8). Immune checkpoint inhibitors (ICIs) such as the PD-1 antibody aim to enhance immune surveillance and control against cancer by releasing the brakes of an antitumor immune response. Currently, ICIs have been investigated in various solid tumors with favorable responses. However, CRC and GC also respond differently to ICI treatment.

The tumor microenvironment (TME) is critical for a patient’s prognosis and drug response. The TME comprises cellular components including endothelial cells, fibroblasts, and immune cells, and non-cellular components such as cytokines, growth factors, hormones, and extracellular matrix, interacting with tumor cells. While the TME plays a critical role in the progress of tumor development from tumor initiation and progression to metastasis, it also has pivotal effects on therapeutic efficacy (9). Thus, the distinct drug response of CRC and GC is likely due to their different tumor microenvironment (TME). Genetic alterations are critical elements in the carcinogenic process and have become one of the significant determinants of the TME (10). In addition, the cross talk between host microbiomes and the TME is also essential. It continuously affects the TME by influencing host immunity and the intestinal epithelium to promote or inhibit the development of tumors (11). A detailed comparison of the TME, genetic landscape, and microbiota of GC and CRC might contribute to a comprehensive understanding of the shaping process of the GC and CRC TME. The TME study needs to exclude the difference in the genetic background of the individual patient, and cancer survivors with second primary malignancies (SPMs) provide such a chance.

SPMs could be divided into synchronous and metachronous malignancies in the same individual, according to the International Agency for Research on Cancer (IARC) (12). Synchronicity was defined as two or more primary malignancies that were diagnosed within 6 months in different sites. In contrast, metachronous malignancies were defined when diagnosis intervals were more than 6 months. Synchronous SPMs were a more suitable system for comparing the TME of the different tumors. About 8.3% of cancer survivors developed an SPM, most of whom died of their second malignancy (13). The incidence of SPMs in adults with gastric cancer (GC) is significantly increased than in the general population (14, 15). Likewise, the patients with colorectal cancer (CRC) were reported to have a higher risk of SPM development (16) and worse survival than patients developing an SPM (17). The synchronous tumor of CRC and GC provides an ideal opportunity to dissect the determinants of distinct TME of CRC and GC in the real world since the genetic background in the same patient is identical.

In the current study, we performed single-cell RNA sequencing (scRNA-seq), WES, and microbiome analysis to depict the TME and dissect the determinants of different TMEs based on the synchronous tumor of CRC and GC. We found that germline mutation created a genetic basis for synchronous CRC and GC, while tumor site-specific mutation and microbiome shaped distinct TMEs.

## Materials and methods

### Single-cell RNA sequencing

The RNA expression of single cells was screened using a 3′ whole transcriptome analysis (WTA) approach through the BD Rhapsody™ WTA Amplification Kit for samples that have been labeled using the BD^®^ Single-Cell Multiplexing Kit for three patients. For each patient of the synchronous patients, pinched biospecimen from CRC, JuxtaCRC, GC, and JuxtaGC sites were digested into single cells with Collagenase D (Roche, 11 088 858 001) and DNase I (Thermo Fisher, 18047019) at 37° for 1 h. Immune cells were enriched *via* a two-layer Percoll (Cytiva, 17-0891-01) density gradient (40% and 70%). For each patient, single-cell solutions from different sites were first barcoded and pooled before library construction. Briefly, the cDNA of mRNA targets is first encoded on BD Rhapsody™ Cell Capture Beads. The barcode information from BD Rhapsody Cell Capture Beads is also added to Sample Tags during reverse transcription, which enables the amplification of Sample Tags in solution. To generate the Sample Tag sequencing libraries, the extended Sample Tags are first denatured from the BD Rhapsody Cell Capture Beads and later amplified through a series of PCR steps. The whole transcriptome amplification library is generated directly from the BD Rhapsody Cell Capture Beads using a random priming approach, followed by an index PCR step. Both the whole transcriptome mRNA and Sample Tag libraries can be combined for sequencing on Illumina sequencers.

### Processing of scRNA-seq data

According to the handbook, sequencing data were aligned toward the human genome (GRCh38) handled by BD Rhapsody™. Briefly, the paired-end FASTQ R1 and R2 files generated from Illumina sequencers were firstly filtered by reading quality. Then, R1 and R2 reads were annotated respectively and combined. Next, the same UMI reads were collapsed into a single raw molecule. The artifact molecules were removed using the Recursive substitution error correction (RSEC) and Distribution-based error correction (DBEC) UMI adjustment algorithms. The putative cells and the sample of origin were further determined.

The output molecule expression matrix was loaded into Seurat (version 4.0.3) (18) for downstream analysis. High-quality cells were kept and processed for normalization and scaling. The top 2,000 highly variable genes were identified using the default parameter. Canonical correlation analysis (CCA) was applied for data integration to remove the batch effect. The integrated data were normalized and scaled. Principal component analysis (PCA) was employed for linear reduction. For visualization, the top 20 principal components (PCs) were selected and fed to the non-linear reduction, Uniform Manifold Approximation, and Projection (UMAP). The clusters were detected using the graph-based clustering algorithm at a resolution of 0.8. Clusters were manually annotated using the typical marker and the top 20 cluster-specific features.

### Whole exome sequencing

For each patient of the these synchronous cancer patients, pinched biospecimen from CRC, GC, and PBMC were collected. DNA was isolated from tissue and PBMC (Qiagen Tissue DNA Kit, 69504), and the quality of isolated genomic DNA was verified using 1% agarose gels and Qubit^®^ DNA Assay Kit in Qubit^®^ 2.0 Fluorometer (Invitrogen, USA). A total amount of 0.6 μg genomic DNA per sample was used as input material for the DNA sample preparation. Sequencing libraries were generated using Agilent SureSelect Human All Exon V6 Kit (Agilent Technologies, CA, USA) following the manufacturer’s recommendations, and index codes were added to each sample. The index-coded samples were performed on a cBot Cluster Generation System using HiSeq PE Cluster Kit (Illumina) for clustering. The DNA libraries were sequenced on the Illumina HiSeq platform, and 150 bp paired-end reads were generated.

### Processing of WES data

The sequence artifacts of raw data were removed. The clean sequencing data were mapped to the reference human genome (UCSC hg19) by Burrows–Wheeler Aligner (BWA) software (19). SAMtools (20) and Picard (http://broadinstitute.github.io/picard/) were employed for duplicate marking, local realignment, and base quality recalibration. SAMtools, mpileup, and bcftools were used to do the variant calling and identify SNP and InDels. ANNOVAR (21) was performed to annotate VCF (variant call format) based on dbSNP, 1000 Genome, and other related existing databases. The somatic SNV was detected by MuTect (22), and the somatic InDel by Strelka (23).

### Microbial DNA extraction and 16S rRNA gene sequencing

For each patient of the these synchronous cancer patients, pinched biospecimen from CRC and GC were collected. Extraction of total genome DNA from samples was extracted using the CTAB method. 16S rRNA/18SrRNA/ITS genes of distinct regions were amplified using a specific primer with the barcode. PCR products were purified with Qiagen Gel Extraction Kit (Qiagen, Germany).

Sequencing libraries were generated using TruSeq^®^ DNA PCR-Free Sample Preparation Kit (Illumina, USA) following the manufacturer’s recommendations, and index codes were added. The library quality was assessed on the Qubit@ 2.0 Fluorometer (Thermo Scientific) and Agilent Bioanalyzer 2100 system. The library was sequenced on an Illumina NovaSeq platform, and 250-bp paired-end reads were generated.

### Processing of OTU data

Paired-end reads were spat by UMI and merged using FLASH (24) for sequence assembly. QIIME (V1.9.1) (25) was applied for data filtration to obtain high-quality clean tags. The UCHIME algorithm (26) was used for chimera removal to get effective tags mapping to the SILVA database (27).

Sequence analysis was performed by UPARSE software (UPARSE v7.0.1001) (28). Sequences with ≥97% similarity were assigned to the same OTUs. For each representative sequence, the SILVA database (27) was used based on the Mothur algorithm to annotate taxonomic information. The alpha diversity analysis was performed using MicrobiomeAnalyst R package.

### Multiplex immunohistochemistry

Opal™ 7-Color Multiplex IHC Kit (Akoya Biosciences, NEL861001KT) was employed to perform multiplex staining. The protocol was referred to the manufacturer’s construction. FFPE sections were incubated at 65°C for at least 18 h as preprocessing. The slide underwent a serial deparaffinization step and then immersed to quench peroxidase followed by washing. The following steps were repeated for multiple-marker staining. The slides were successively treated for primary antigen retrieval, blocking of unspecific binding, secondary antibody conjugating, and stripping. After completing multiple stainings, the slides were scanned on the PerkinElmer Vectra 3^®^ Polaris™ platform and imaged on the inForm Advanced Image Analysis software (inForm 2.4.1; Akoya Biosciences, USA) with the DAPI (Akoya Biosciences) filter set. The antibodies and reagents are listed in **Table S1**.

### Statistical analysis

Additional statistical analysis was performed in R software (version 4.1.1). The ggplot2 package was used for visualization. In addition, the R package “Survminer” was implemented to analyze survival differences between different groups. Statistical tests were selected based on the appropriate assumptions for the data distribution and variability characteristics. Sample data were analyzed by a two-tailed Student’s t-test to identify statistically significant differences between CRC and GC groups. One-way ANOVA with the Bonferroni posttest was used to identify differences among the GC, CRC, JuxtaCRC, and JuxtaGC groups. A p-value less than 0.05 was considered statistical significance.

## Results

### scRNA-seq reveals the distinct features of TME between CRC and GC

**Figure 1:**
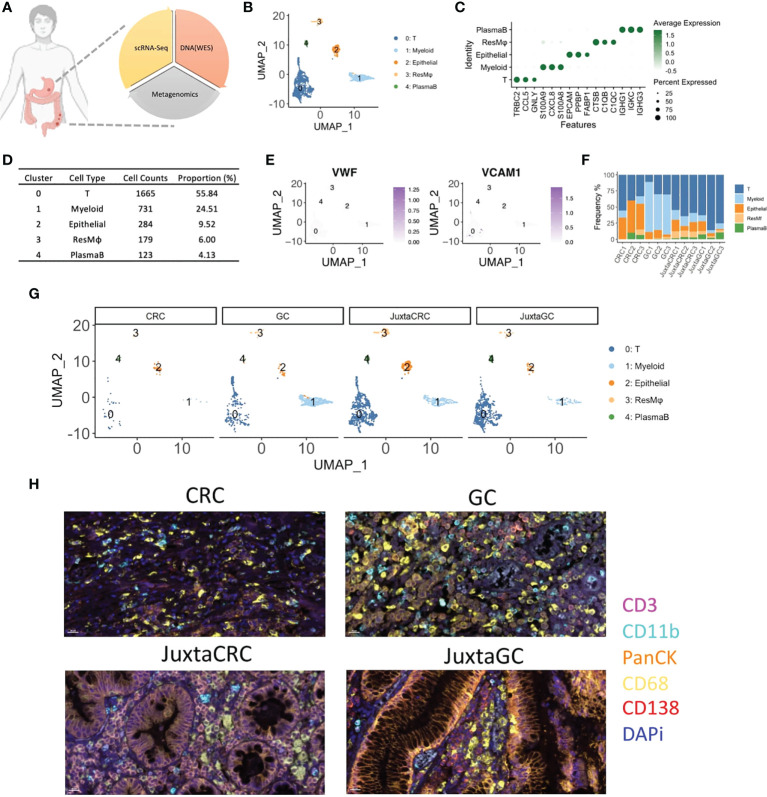
The overview of scRNA-seq profiles of CRC and GC. **(A)** The schematic of experimental design in this study. ScRNA-seq, WES, and OTU investigated the primal malignancy sites’ transcriptomics, genomics, and metagenomics profile. **(B)** UMAP plot of scRNA-seq data identified five cell populations, including T cells, myeloid cells, epithelial cells, residential macrophages (ResMφ), and plasma B cells. **(C)** Dot plot showed the top 3 cluster-specific feature genes, dot size indicated the percent expressed, and the level of color indicated the average expression. **(D)** The distribution frequency and proportion of each cell population. **(E)** Feature plots showed the expression pattern of the diagnostic marker genes for endothelial cells, including VWF and VCAM1. **(F)** Bar plot displayed the proportion of each cell population across samples. **(G)** UMAP plots of scRNA-seq data spat by samples. **(H)** mIHC staining of CD3 (magenta), CD11b (cyan), PanCK (orange), CD68 (yellow), CD138 (red), and DAPi (blue) in CRC, GC, JuxtaCRC, and JuxtaGC sites.

The malignancy (CRC and GC) and adjacent normal (JuxtaCRC and JuxtaGC) samples were analyzed by single-cell RNA sequencing, whole-exome sequencing (WES), and microbiome sequencing to cover cellular mutations and microbiome components of the TME and gain a comprehensive understanding of the heterogeneity of a dual-malignancy tumor immune signature (**Figure 1A**). Moreover, the clinical details of patients enrolled are listed in **Table S2**.

After single-cell RNA sequencing, 2,982 cells passed the quality control threshold (minimum of 200 genes, maximum of 6,000 genes, and <20% mitochondrial reads per cell), selected for the downstream analysis. scRNA-seq data revealed five main cell populations characterized by the typical markers (**Figure 1B**). The majority of cells (cluster 0: 1,665 cells with a proportion of 55.84%) were defined as T cells characterized by the expression of T-cell receptor beta constant 2 (TRBC2) (**Figures 1C, D**). The high expression of calprotectin (S100A9 and S100A8) and chemokines (CXCL8) confirmed the presence of myeloid cells (cluster 1). Epithelial cells (cluster 2) were defined by the epithelial cell adhesion molecule (EPCAM) gene. Macrophages (cluster 3) were characterized by the genes engaged in complement activation (C1QA and C1QB). Cluster 4 harboring the genes associated with immunoglobulin (IGHG1, IGKC, and IGHG3) was annotated as plasma B cells. The frequency of each population is displayed in **Figure 1D**.

Our cell isolation procedures mainly purified immune cells. Thus, stromal cells were missing in our scRNA-seq profiling of the TME. Stromal cells like endothelial cells (VWF^+^ and VAM^+^) were not identified (**Figure 1E**).

The distribution of each population across samples is shown in **Figures 1F, G**. We found that the components of adjacent normal samples (JuxtaCRC and JuxtaGC) were analogous. T cells hold a dominant position, and the proportion of granulocytes, epithelial cells, macrophages, and plasma B cells was comparable, which were found in both JuxtaCRC and JuxtaGC. Compared to the corresponding adjacent normal sample, there was a significant enrichment of immune cells in the GC sample. Notwithstanding, the macrophages and myeloid cells were dramatically increased in the GC sample, showing the diversity of the TME between CRC and GC. All the above results were validated by mIHC staining as well (**Figure 1H**).

Based on a detailed dissection of TME cellular components *via* scRNA-seq, we could observe a similar cellular distribution pattern between adjacent normal samples of GC and GRC, originating from the same genetic background. However, even based on the same genetic background, the TMEs of GC and CRC were quite different, indicating other cofounding factors of TME determination, such as mutational profiles and microbiome.

### T-cell subsets enrichment in GC

**Figure 2:**
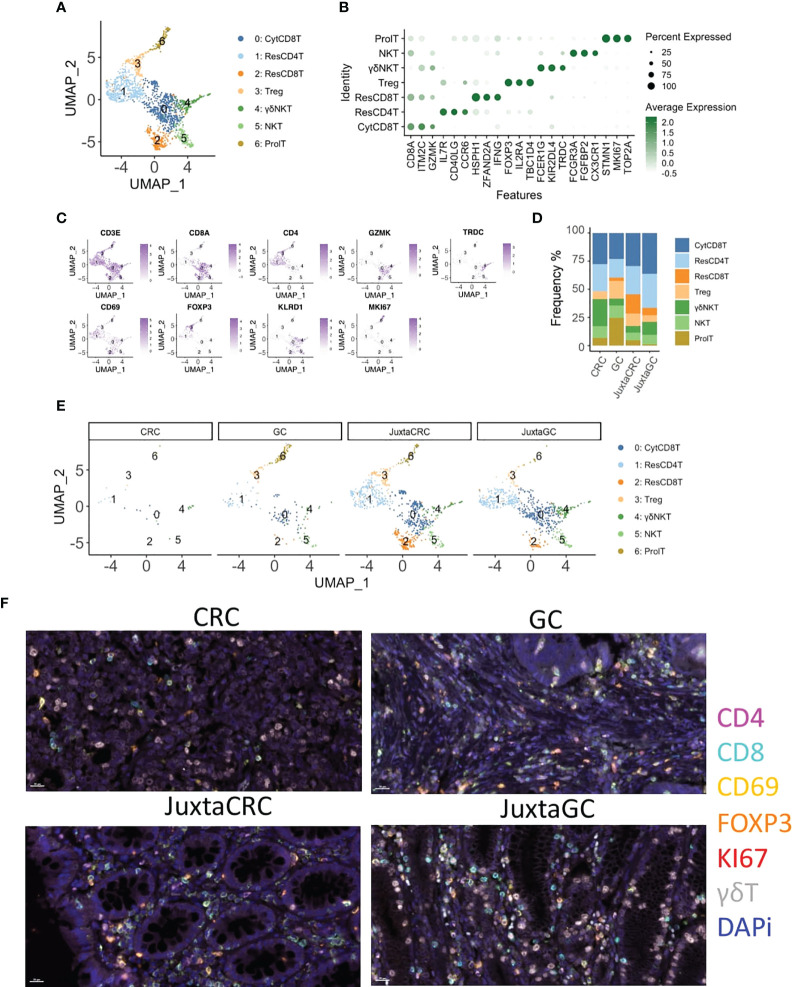
T-cell diminishment in CRC. **(A)** UMAP plots of the T-cell population reveal seven cell subsets, namely, cytotoxic CD8^+^ T cells (CytCD8T), CD4 T cells (ResCD4T), resident CD8+ T cells (ResCD8T), regulatory T cells (Treg), gamma delta NK T cells (γδNKT), NK T cells (NKT), and proliferating T cells (ProlT). **(B)** Dot plot shows the top three cluster-specific feature genes, dot size indicates the percent expressed, and the level of color indicates the average expression. **(C)** Feature plots show the expression pattern of the diagnostic marker genes for cluster annotation, including CD3E (T cells), CD8A (CD8^+^ T cells), CD4 (CD4^+^ T cells), GZMK (CD8^+^ T cells), TRDC (gamma delta T cells), CD69 (resident T cells), FOXP3 (Treg), KLRD1 (NK cells), and MKI67 (proliferating cells). **(D)** Bar plot displays the population of each T-cell subset across samples. **(E)** UMAP plots of the T-cell population spat by samples. **(F)** mIHC staining of CD4 (magenta), CD8 (cyan), CD69 (yellow), FOXP3 (orange), KI67 (red), γδT (white), and DAPi (blue) in CRC, GC, JuxtaCRC, and JuxtaGC sites.

Next, we investigated the T-cell population to see the T-cell subset difference between GC and CRC. A refined UMAP analysis was performed. The in-depth clustering analysis revealed seven refined subsets (**Figures 2A, B**), including 2 CD8^+^ T-cell subsets, cytotoxic CD8^+^ T cells (CytCD8T, cluster 0), and resident CD8+ T cells (ResCD8^+^ T, cluster 2), distinguished by the expression of GZMK and CD69 (29). Cluster 1 expressed CD69 and CD4 simultaneously. Thus, cluster 1 was defined as resident CD4 T cells (ResCD4T). The other CD4 positive T-cell subset was annotated as regulatory T cells (Treg, cluster 3) according to the expression of the Treg-specific marker, FOXP3. Clusters 4 and 5 that expressed both T-cell marker CD3E and NK cell marker KLRD1/FCGR3A were recognized as NK T cells (NKT), and cluster 4 was further identified as gamma delta NK T cells (γδNKT) based on the presence of TRDC. The marker of proliferation, Ki-67 (MKI67), was used to define proliferating T cells (ProlT, cluster 6) (**Figure 2C**).

The UMAP plot highlighted by samples implied that the majority of the T population was contributed by JuxtaCRC, followed by JuxtaGC (**Figures 2D, E**). Moreover, both JuxtaCRC and JuxtaGC shared similar cellular components (**Figure 2E**). T-cell diminishment was observed in CRC compared with T-cell subsets in corresponding normal tissue (**Figure 2E**). In addition, regulatory T cells (Treg) and proliferating T cells (ProlT) were detected in GC (**Figure 2E**).

Overall, we could observe that T-cell subsets predominated in the juxta-tumoral normal site and the distribution pattern of T-cell subsets was also similar between adjacent areas of GC and CRC. However, the distribution of T-cell subsets within GC and CRC tumoral sites was distinct. T-cell subsets were diminished in CRC while the GC site was enriched for Treg and proliferating T cells (**Figure 2F**).

### Myeloid cell enrichment in GC

**Figure 3:**
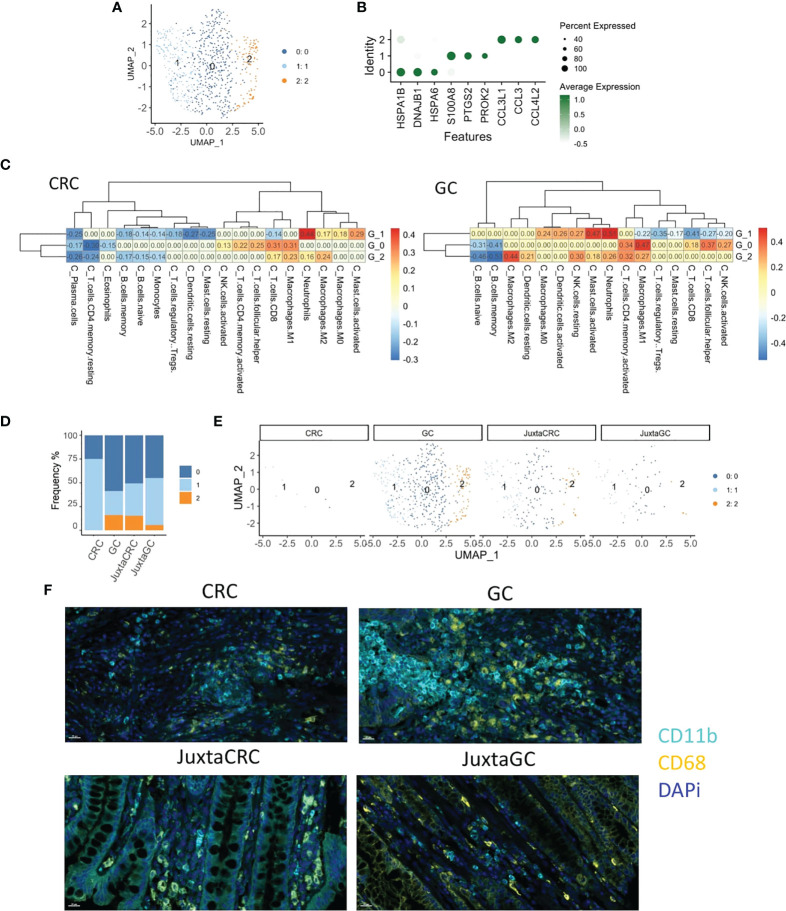
Myeloid cell enrichment in the GC site. **(A)** UMAP plots of the myeloid cell population reveal three cell subsets, namely, clusters 0, 1, and 2. **(B)** Dot plot shows the top three cluster-specific feature genes, dot size indicates the percent expressed, and the level of color indicates the average expression. **(C)** Heatmaps show the correlation relationship between subset GSVA score and the fraction of 22 subpopulations of immune cells of CRC and GC, respectively, generated by CIBERSORT (left: CRC, right: GC). **(D)** Bar plot displays the population of each myeloid cell subset across samples. **(E)** UMAP plots of the myeloid cell population spat by samples. **(F)** mIHC staining of CD11b (cyan), CD68 (yellow), and DAPi (blue) in CRC, GC, JuxtaCRC, and JuxtaGC sites.

To explore the heterogeneity of myeloid cells in GC and CRC, we conducted an in-depth UMAP analysis. More narrowly, the myeloid population was subdivided into three clusters (**Figure 3A**), HSP^+^ cluster 0, S100A8^+^ cluster 1, and CCL3/4^+^ cluster 2 (**Figure 3B**).

To investigate the detailed cell identification of different clusters in the myeloid lineage, we explored the relationship between myeloid subsets and tumor-infiltrating leukocytes (TILs). The TIL frequency was estimated by the Cell-type Identification by Estimating Relative Subsets of RNA Transcripts (CIBERSORT) algorithm (30) based on bulk tissue gene expression profiling of CRC and GC extracted from The Cancer Genome Atlas (TCGA).

The fraction of 22 subpopulations of immune cells per sample was calculated using the CIBERSORT algorithm. Samples with a p-value no less than 0.05 were removed. The correlation test was performed on CRC and GC, respectively, between the immune components of bulk RNA-seq and GSVA scores of three myeloid subsets in scRNA-seq.

Cluster 0 exhibited the highest correlation with M1 macrophage, implying that cluster 0 harbored a similar phenotype to M1 macrophage. The other phenotype of macrophage M2 was identified as the cluster 2-associated immune type. The most substantial relationship between cluster 1 and neutrophils suggested that the constituents of cluster 1 were neutrophil-like cells. Myeloid cell identity was confirmed on CRC and GC datasets (**Figure 3C**).

We could observe that most myeloid cells were identified in the GC site (**Figures 3D, E**), while myeloid cells were minimal in the CRC site. In addition, myeloid cells in juxta-tumoral sites were also less than at the GC site (**Figures 3D, E**), indicating that myeloid cells were mainly recruited into the GC site. Moreover, the distribution pattern of myeloid cells were also verified by mIHC staining (**Figure 3F**).

### Specific epithelial subset enrichment in different TMEs

**Figure 4:**
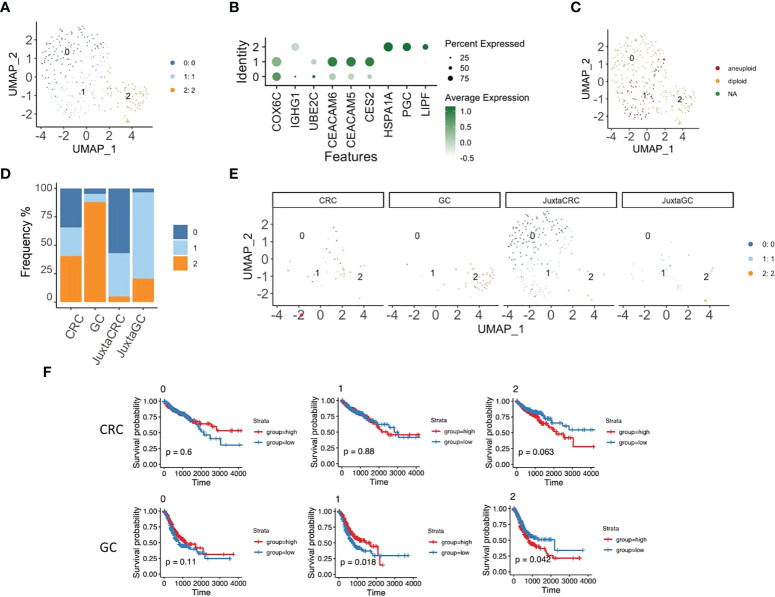
Specific epithelial subset enrichment in different TMEs. **(A)** UMAP plots of the epithelial cell population reveal three cell subsets, namely, clusters 0, 1, and 2. **(B)** Dot plot shows the top three cluster-specific feature genes, dot size indicates the percent expressed, and the level of color indicates the average expression. **(C)** UMAP plot of epithelial cell population highlighted by copyKAT results; red refers to aneuploid, yellow refers to diploid, and green refers to NA. **(D)** Bar plot displays the population of each epithelial cell subset across samples. **(E)** UMAP plots of epithelial cell population spat by samples. **(F)** Kaplan–Meier plots show the survival results of the epithelial cell subset GSVA score for CRC and GC based on TCGA database. Red refers to the high group, and blue refers to the low group (top panel: CRC, bottom panel: GC).

Next, we took a deeper insight into the epithelial cell population to illustrate the distribution of malignant cells. The reclustering analysis revealed three subsets of epithelial cells (**Figures 4A, B**).

We first performed copyKAT (31) to identify malignant cells, and only epithelial subset 1 had aneuploid malignant cells (**Figure 4C**). Also, epithelial subset 1 was enriched in the CRC site, while diploid epithelial subsets 0 and 2 were increased in the juxta-CRC and GC sites, respectively (**Figures 4D, E**). Surprisingly, the dominant epithelial subset 2 in GC was diploid (**Figures 4C, D**).

In addition, we dissected the relationship of each epithelial subset to the patient’s prognosis. The GSVA score for each epithelial subset was calculated using bulk RNA-Seq data from TCGA based on epithelial subset feature genes (**Table S3**). Moreover, the correlation between the epithelial subset-specific GSVA score and patients’ prognosis was investigated. We found that the survival time was shorter in the high group than in the low group based on the GSVA score of epithelial subset 2 in GC and CRC patients (**Figure 4F**). We also observed that a higher frequency of aneuploid epithelial subset 1 was not associated with a worse prognosis. The higher frequency of aneuploid epithelial subset 1 indicated a better prognosis in the GC patients (**Figure 4F**), probably due to the better neoantigen generation based on a higher mutational burden.

Overall, we noticed distinct epithelial subset enrichment in GC and CRC subsets, possibly due to different mutation profiles between GC and CRC (as described below). Moreover, it seems that aneuploid malignant cells are not necessarily linked to worse prognosis in GC patients since aneuploid malignant cells could be associated with higher levels of antigen generation.

### Different mutational profiles lead to distinct drug responses of CRC and GC

**Figure 5:**
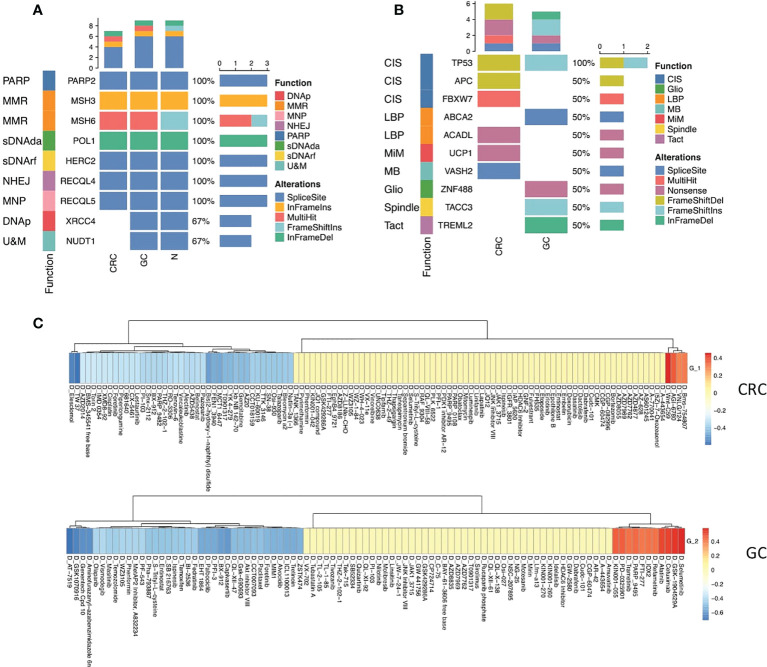
Mutational profiles of GC and CRC. **(A)** Oncoplot shows the germline mutations in CRC, GC, and normal sites. The top bar shows the counts of alterations type in each sample. The left annotation indicated the functions of the genes. The right bar shows the counts of alteration type of each gene. **(B)** Oncoplot shows the somatic mutations in CRC and GC sites. The top bar shows the counts of alteration type in each sample. The left annotation indicates the functions of the genes. The right bar shows the counts of alteration type of each gene. **(C)** Heatmaps show the correlation relationship between the subset GSVA score and the drug sensitivity for CRC and GC, respectively, collected from the GDSC database (top panel: CRC, bottom panel: GC).

To gain insights into the genetic alterations associated with the pathogenesis and drug response of GC and CRC, we performed whole-exome sequencing of GC, CRC, and the corresponding juxta-tumoral normal samples. A total of nine DNA repair-associated susceptible genes (PARP2, MSH3, MSH6, XRCC4, NUDT1, POLI, HERC2, RECQL4, and RECQL5) were detected as germline mutations (**Figure 5A**), which engaged in poly(ADP-ribose) polymerase (PARP) enzymes that bind to DNA (PARP), mismatch excision repair (MMR), non-homologous end-joining (NHEJ), modulation of nucleotide pools (MNP), DNA polymerases (DNAp), ubiquitination and modification (U&M), diseases associated with sensitivity to DNA damaging agents (sDNAda), and other known or suspected DNA repair function (sDNArf). Seven out of nine genes (PARP2, MSH3, MSH6, POLI, HERC2, RECQL4, and RECQL5) alternated in CRC and GC. The pattern of alternations was consistent, indicating that multiple alternations in DNA repair genes were likely to be the oncogenic divers of synchronous malignancies. The other two genes (XRCC4 and NUDT1) were only detected in GC. Moreover, we noticed that splice site was the dominant target of mutation.

Somatic mutation analysis revealed that the top 10 genes (TP53, APC, FBXW7, ABCA2, ACADL, UCP1, VASH2, ZNF488, TACC3, and TREML2) were rearranged in the site of two primary malignancies that participated in chromosomal instability (CIS), Wnt signaling pathway (WNT), negative regulation of lipid biosynthetic process (LBP), mitochondrial inner membrane (MiM), microtubule-binding (MB), gliogenesis (Gilo), spindle (Spindle), and T-cell activation (TAct) (**Figure 5B**, **Figure S1**).

TP53 is a critical tumor-suppressor gene, one of the most frequent somatic alterations in human cancer. The alternation of the TP53 gene was found in both CRC and GC. Nevertheless, the mutation pattern was distinct. Frameshift deletions occurred in CRC, and frameshift insertions were detected in GC, reflecting that the mutation of the TP35 gene was vital to both CRC and GC tumor initiation (**Figure 5B**).

Five mutated somatic genes were specific to CRC, namely, APC, FBXW7, ACADL, UCP1, and VASH2, while GC was characterized by the remaining four genes, which were ABCA2, ZNF488, TACC3, and TREML2. Interestingly, inactivating mutations in the APC gene merely appeared in CRC, which has been reported as a critical genetic factor in the initiation and progression of CRC (32), demonstrating that the loss of APC may be one of the main driving forces of CRC tumorigenesis in synchronous malignancies. Additionally, the somatic mutation of APC as a regulator of chromosome integrity (33) may contribute to developing aneuploid epithelial subset 1 enrichment in the TME of CRC (**Figure 5B**).

Next, we deciphered the role of epithelial subsets in drug response, and the connection between drug sensitivity and three epithelial subsets was analyzed. The RNA-sequencing results of an extensive collection of GC and CRC cell lines and AUC values of drugs to the GC and CRC cell lines representing the overall drug effect on each cell line were obtained from the Genomics of Drug Sensitivity in Cancer (GDSC) database (34). The GSVA score of each subset was calculated using the top 20 subset-specific genes (**Table S3**) using the RNA-sequencing results of each cell line. Interestingly, aneuploid epithelial subset 1 enriched in the TME of CRC was highly susceptible to the APC inhibitor, consistent with the APC mutation detected specifically in CRC, while epithelial subset 2 enriched in the TME of CRC was sensitive to kinase inhibitors (**Figure 5C**).

Our findings revealed common and distinct mutational profiles underlining the CRC and GC. The common mutation in DNA repair and p53 pathways could contribute to the genetic background of synchronous tumor initiation. Moreover, distinct mutation profiles might explain the specific cellular subset distribution of GC and CRC. Particular mutation profiles also contributed to different drug responses.

### DNA repair and metabolism-related microbiome in the CRC and GC TME

**Figure 6:**
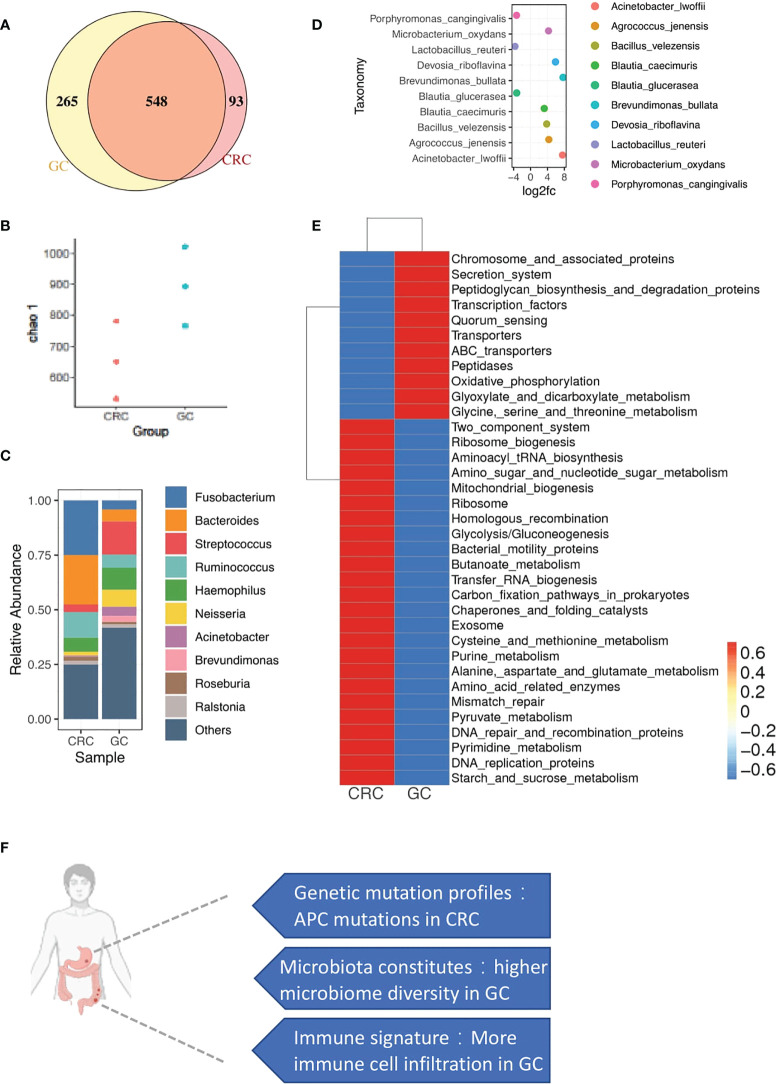
microbial profiles of CRC and GC. **(A)** Venn diagram shows the overlap of microbiotas from CRC and GC. Five hundred forty-eight microbiotas were shared between GC and CRC. Two hundred sixty-five microbiotas unique to GC, 93 microbiotas unique to CRC. **(B)** Plot shows the chao 1 alpha diversity index distribution between CRC and GC groups. **(C)** Bar plot shows the relative abundance of the top 10 microbiotas in CRC and GC at the genus level. **(D)** Dot plot shows the top 10 differentially abundant OTUs between CRC and GC at the species level. **(E)** Heatmap shows the function enrichment pathway of microbiotas in CRC and GC. **(F)** A brief summary of major differences between CRC and GC in synchronous cancer patients.

To investigate the diversity of microbial communities between CRC and GC sites, we performed 16S rRNA gene sequencing. In total, 1,454 variants were identified at the level of genus. Five hundred forty-eight microbiotas are commonly microbially detected in CRC and GC. Such similarity might result from the similar origin of intestinal locations. The primary malignancy-specific microbiotas were also observed, 93 in CRC and 265 in GC (**Figure 6A**). We also observed that microbiota diversity was much higher in the GC site (**Figure 6B**).

We performed a closer examination to gain a deeper insight into the community composition in synchronicity tumors. The top 10 most abundant microbiotas are displayed in **Figure 6C** *Fusobacterium*, *Bacteroides*, and *Ruminococcus* were highly enriched in CRC. *Streptococcus*, *Acinetobacter*, and *Brevundimonas* were dominated in GC (**Figures 6C, D**). In gut microbe studies, Fusobacterium has been reported as a dominant genus associated with CRC (35, 36).

Functional characteristics of microbiotas were further assessed through Tax4Fun (37). We noticed that the GC microbiotas were related to central carbon and amino acid metabolism pathways (**Figure 6E**), such as glyoxylate and dicarboxylate metabolism and glycine/serine/threonine metabolism (**Figure 6E**). Interestingly, GC sites were enriched with immunes. Immune-metabolism connection was consistent with the previous report that immune cells, rather than the tumor cells in the TME, carried high metabolic activity (38). Consistent with the WES and copyKAT results, DNA repair-associated microbiota were also observed in CRC, probably related to the enrichment of aneuploid malignant cells (**Figure 6E**), including mismatch repair, DNA repair, and recombination proteins and DNA replication proteins.

Overall, we found the specific distribution of microbiota in GC and CRC with different functions. Moreover, the functional features of microbiota were consistent with the cellular and mutational landscapes of the GC and CRC TME.

## Discussion

We utilized the same genetic background of the synchronous primary tumor to comprehensively illustrate the TME of CRC and GC *via* scRNA-seq, WES, and microbiome analyses. We found that the cellular components of the juxta-tumoral sites of CRC and GC are very similar. CRC was mainly composed of aneuploid malignant cells, and GC was composed of diploid epithelial cells, Tregs, and myeloid suppressor cells. Germline mutation in the DNA repair process provides the genetic background of the synchronous GC and CRC. Moreover, chromosome integrity mutation was specific in the CRC site, corresponding to the aneuploid malignant cells identified in the CRC. Microbiomes related to the metabolism were enriched in the GC, consistent with the infiltration of immune cells in GC. Furthermore, the microbiome-related DNA repair process was enriched with chromosome integrity mutations in the CRC. Overall, the mutational landscape and microbiome determine the TME together (**Figure 6F**).

Calprotectin (S100A9 and S100A8) and chemokines (CXCL8) expressing myeloid cells were also identified in other scRNA-seq studies about infectious diseases (39) and tumors (40). S100A8/9^+^ myeloid cells were reported to recruit neutrophils (41). In addition, S100A8/9^+^ myeloid cells could promote angiogenesis and exacerbate cancer through the S100a8/S100a9–Emmprin–Vegfa axis (42).

Macrophages were characterized by the genes engaged in complement activation (C1QA and C1QB). C1QA/B^+^ macrophages were often referred to as resident macrophages in several scRNA-seq profiling studies for different types of cancer (43, 44). It has been shown that tumor cells could hijack macrophage-produced C1QA/B proteins to promote tumor growth (45).

APC mutation could be identified in both GC and CRC (46, 47). Our current study detected APC mutation specifically in the CRC site. Moreover, APC mutation was associated with the cold tumor (48). Thus, the immune dessert phenotype of CRC was due to the APC mutation. The GC TME was enriched with myeloid suppressor cells, including macrophages and neutrophils. Consistently, the Treg frequency was also higher in GC, probably due to the immune inhibitory function of myeloid suppressors (49). We also found that *Brevundimonas* was enriched in gastric cancer with more Tregs, consistent with the previous report that regulatory T cells within the tumor microenvironment in gastric cancer were correlated with gastric microbiota (50).

### Limitations of the study

Our analysis mainly compared normal/adjacent tissue with full-blown cancer. Thus, we might miss the critical preventative treatment targets on non-T immune cells and other cell types in the tumor microenvironment, such as stromal cells. In addition, the stromal cells might be missing during our cell isolation procedures, which was designed for immune cell enrichment, while stromal cells were also essential players of the TME and missing such cells were significant limitations of our current study (51).

## Conclusions

Based on the same genetic background of the synchronous tumor, GC and CRC TME were dissected comprehensively. We found that the TME was mainly determined by the mutational landscape and microbiome, which shed light on future combinational therapy targeting the mutation and microbiome together.

## Data availability statement

The data presented in the study are deposited in the GSA-human repository, accession number HRA002301, HRA002299 and HRA002300. 

## Ethics statement

The studies involving human participants were reviewed and approved by the Ethics Committee of Tianjin Medical University. The patients/participants provided their written informed consent to participate in this study.

## Author contributions

Conceptualization, WY, YZ, QG, XW, JS and XC; Methodology, WY, YZ, QG, XW and JS; Investigation, WY, YZ, QG, XW, YJ, JZ, GL, FC, XW, YY and JS; Writing-Original Draft Preparation, WY, YZ and JS; Writing-Review & Editing, QG, JZ, HH, XL, JD, JS and XC; Supervision, JS and XC; Project Administration, QG; Funding Acquisition, HH, JD and JS. All authors contributed to the article and approved the submitted version.

## Funding

This work was supported by the National Key Research and Development Program of China (grant 2019YFA0803000 to JS), the Excellent Youth Foundation of Zhejiang Scientific (grant R22H1610037 to JS), the National Natural Science Foundation of China (grant 82173078 to JS), the Natural Science Foundation of Zhejiang Province (grant 2022C03037 to JS)., the National Key Research and Development Project of China (Grant No. 2019YFA0905600 to HH), the National Natural Science Foundations of China (82000489 to JD), Zhejiang Provincial Natural Science Foundation (LY19H030010 to JD).

## Conflict of interest

The authors declare that the research was conducted in the absence of any commercial or financial relationships that could be construed as a potential conflict of interest.

## Publisher’s note

All claims expressed in this article are solely those of the authors and do not necessarily represent those of their affiliated organizations, or those of the publisher, the editors and the reviewers. Any product that may be evaluated in this article, or claim that may be made by its manufacturer, is not guaranteed or endorsed by the publisher.

## Supplementary material

Supplementary Table 1 List of antibodies

Supplementary Table 2 Clinical information of patients enrolled

Supplementary Table 3 The top 20 feature genes of epithelial subsets

Supplementary Figure 1 Mutational profiles of GC and CRC. **(A)**: Oncoplot showed the somatic mutations in each of the CRC and GC sites. The top bar showed the counts of alterations type in each sample. The left annotation indicated the functions of the genes. The right bar showed the counts of alterations type of each gene. **(B)**: Mutations detected in more than one of the CRC and GC sites were clustered together.

## Abbreviations

scRNA-seq, single-cell RNA-sequencing; mIHC, multiplex immunohistochemistry; CRC, colorectal cancer; GC, gastric cancer
