# Supplementary material for: Genetic mutation and tumor microbiota determine heterogenicity of tumor immune signature: Evidence from gastric and colorectal synchronous cancers
Source: Front Immunol. 2022 Nov 7;13:947080. doi: 10.3389/fimmu.2022.947080 (PMC9676241; doi:10.3389/fimmu.2022.947080)
Supplement: Supplementary Table 1 — List of antibodies [file DataSheet_1.pdf]

List of antibodies

Antibodies	Source	Identifier
CD3	Cell signaling technoiogy	Cat#: 85061s
CD11b	abcam	Cat#: ab13357
Pan cytokeratin	biolegend	Cat#: 914704
CD68	biolegend	Cat#: 916104
CD138	invitrogen	Cat#: 362900
CD4	abcam	Cat#: ab133616
CD8	biolegend	Cat#: 372902
CD69	abcam	Cat#: ab234511
Foxp3	Cell signaling technoiogy	Cat#: 12653s
Ki67	BD	Cat#: 556609
GAMMADELTA	Novus Biologicals	Cat#: NBP2-62225
