# Supplementary material for: Genetic mutation and tumor microbiota determine heterogenicity of tumor immune signature: Evidence from gastric and colorectal synchronous cancers
Source: Front Immunol. 2022 Nov 7;13:947080. doi: 10.3389/fimmu.2022.947080 (PMC9676241; doi:10.3389/fimmu.2022.947080)
Supplement: Supplementary Table 2 — Clinical information of patients enrolled [file DataSheet_3.pdf]

Clinical informat

Sample Number	pathologic diagnoses	Gender	Age(y/o)	Location
1	moderately-poorly differentiated	M	69	colon (patient 1)
2	well-moderately differentiated	F	68	colon (patient 2)
3	well-moderately differentiated	M	31	colon (patient 3)
4	poorly differentiated	M	69	gastric (patient 1)
5	poorly differentiated	F	68	gastric (patient 2)
6	poorly differentiated	M	31	gastric (patient 3)

ion of patients enrolled

Gross appearance	Maximum Diameter(mm)	pTNM Classification	Staging	vascular invasion	omentum invasion
Ulcerative	30	T4aN1bM0	IIIB	-	-
Ulcerative	30	T4aN0M0	IIB	-	-
Ulcer, everted edge	50	T4aN0M0	IIB	-	-
Ulcerative	60	T4N2M0	IIIA	+	-
Extraluminal	40	T4aN2M0	IIIA	-	-
Ulcerative	20	T4aN1M0	IIIA	-	-

perineural invasion	lymphatic metastasis	note	**Immunohistochem**	
			MSH2	MSH6
-	+	/	+	+
-	-	/	+	+
-	-	partly mucinous adenocarcinoma	+	+
+	+	/	+	+
+	+	partly signet ring cell	+	+
+	+	partly signet ring cell	+	+

nistry

PMS2	MLH1	Ki67	HER2	ERER	CK7
+	+	/	/	/	/
+	+	/	/	/	/
+	+	70%	/	/	/
+	+	/	-	-	+
+	+	/	-	-	+
+	+	80%	-	-	+
