# Supplementary material for: Genetic mutation and tumor microbiota determine heterogenicity of tumor immune signature: Evidence from gastric and colorectal synchronous cancers
Source: Front Immunol. 2022 Nov 7;13:947080. doi: 10.3389/fimmu.2022.947080 (PMC9676241; doi:10.3389/fimmu.2022.947080)
Supplement: Supplementary Table 3 — The top 20 feature genes of epithelial subsets [file DataSheet_2.pdf]

The top 20 feature genes of epithelial subsets

	cluster	gene
1	0	RPS21
2	0	TARS
3	0	RPL7
4	0	SMC2
5	0	HJURP
6	0	RPL12
7	0	GNG4
8	0	LGALS3
9	0	ALDOC
10	0	COX6C
11	0	PTTG1
12	0	IGHG1
13	0	CEACAM7
14	0	PAICS
15	0	UBE2C
16	0	PRELID3B
17	0	RAN
18	0	TMEM45B
19	0	PPBP
20	0	SELENBP1
21	1	ERBB3
22	1	APP
23	1	CLDN1
24	1	CAMK2N1
25	1	CCT3
26	1	CEACAM6
27	1	CD24
28	1	SLC11A2
29	1	CEACAM5
30	1	ATP1B1
31	1	CES2
32	1	SCD
33	1	CLDN4
34	1	GPX2
35	1	H1F0
36	1	ALDOB
37	1	SLC12A2
38	1	LCN2
39	1	LYZ
40	1	DMBT1
41	2	ACTA2
42	2	TAGLN
43	2	CALD1
44	2	HSPA1A

45	2	MUC4
46	2	GBP5
47	2	PGC
48	2	FOSB
49	2	NR4A1
50	2	JCHAIN
51	2	RGS2
52	2	IGHA2
53	2	CXCR4
54	2	SRGN
55	2	HSPA1B
56	2	DNAJB1
57	2	PPP1R15A
58	2	TIMP2
59	2	LIPF
60	2	ITGAX
