# Supplementary figures and images for: Genetic mutation and tumor microbiota determine heterogenicity of tumor immune signature: Evidence from gastric and colorectal synchronous cancers
Source: Front Immunol. 2022 Nov 7;13:947080. doi: 10.3389/fimmu.2022.947080 (PMC9676241; doi:10.3389/fimmu.2022.947080)

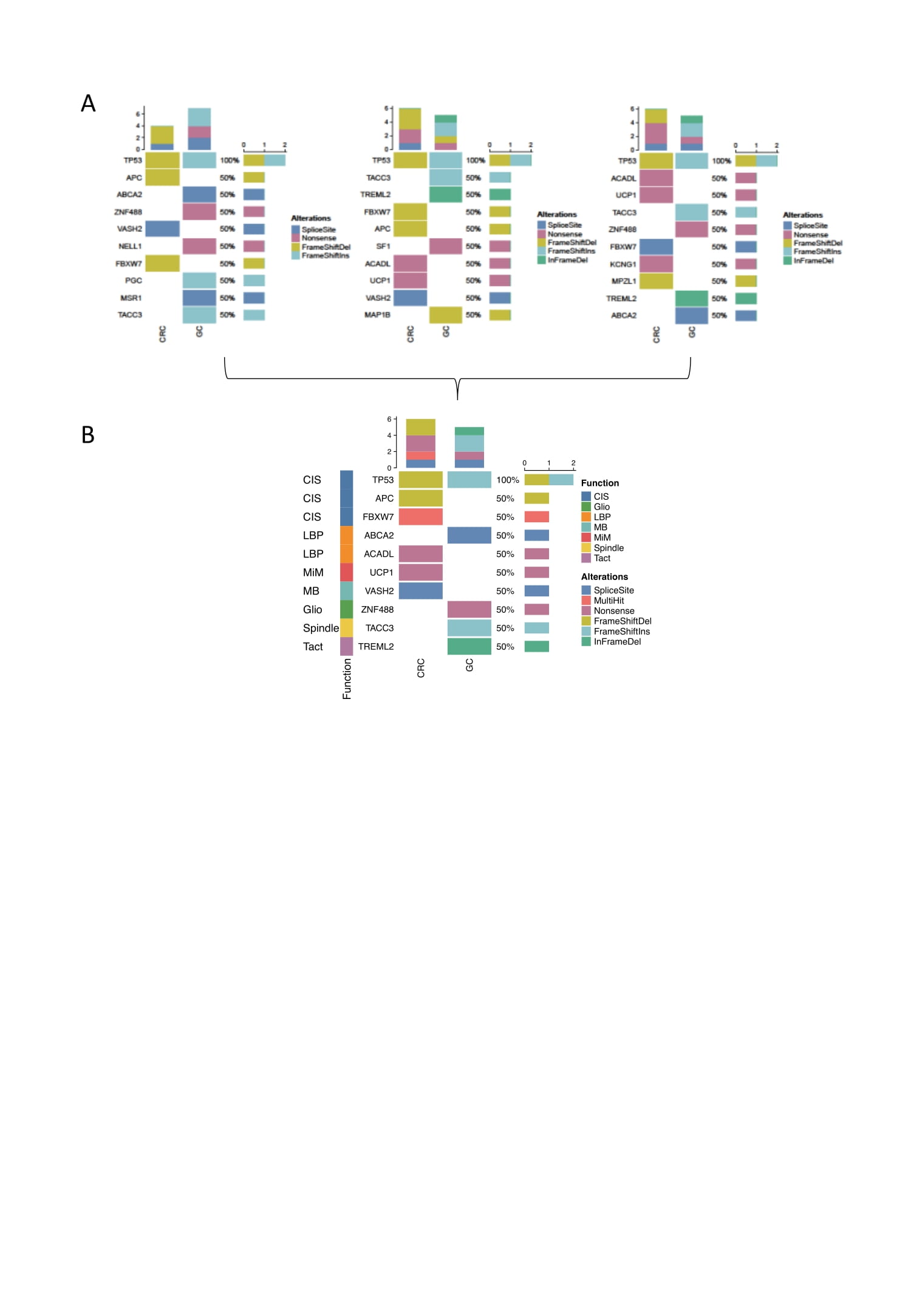

Supplement: Supplementary Figure 1 — Mutational profiles of GC and CRC. (A): Oncoplot showed the somatic mutations in each of the CRC and GC sites. The top bar showed the counts of alterations type in each sample. The left annotation indicated the functions of the genes. The right bar showed the counts of alterations type of each gene. (B): Mutations detected in more than one of the CRC and GC sites were clustered together. [file Image_1.jpeg]
